# Fluorescent Probes for Insect Ryanodine Receptors: Candidate Anthranilic Diamides

**DOI:** 10.3390/molecules19044105

**Published:** 2014-04-02

**Authors:** Yi Wang, Lei Guo, Suzhen Qi, Hao Zhang, Kechang Liu, Ruiquan Liu, Pei Liang, John E. Casida, Shangzhong Liu

**Affiliations:** 1 Department of Applied Chemistry, College of Science, China Agricultural University, No. 2 Yuanmingyuan West Road, Beijing 100193, China; E-Mails: royalwy@gmail.com (Y.W.); forgoodbaby@163.com (S.Q.); cauzhanghao@gmail.com (H.Z.); lucklkc@163.com (K.L.); fengzilrq@163.com (R.L.); 2 College of Agriculture and Biotechnology, China Agricultural University, No. 2 Yuanmingyuan West Road, Beijing 100193, China; E-Mails: guoleicau@hotmail.com (L.G.); liangcau@cau.edu.cn (P.L.); 3 Department of Environmental Science, Policy, and Management, University of California, Berkeley, CA 94720-3112, USA; E-Mail: ectl@berkeley.edu

**Keywords:** fluorescent probe, affinity, ryanodine receptor

## Abstract

Diamide insecticides with high efficacy against pests and good environmental safety are broadly applied in crop protection. They act at a poorly-defined site in the very complex ryanodine (Ry) receptor (RyR) potentially accessible to a fluorescent probe. Two *N*-propynyl analogs of the major anthranilic diamide insecticides chlorantraniliprole (Chlo) and cyantraniliprole (Cyan) were accordingly synthesized and converted into two fluorescent ligands by click reaction coupling with 3-azido-7-hydroxy-2*H*-chromen-2-one. The new diamide analogs and fluorescent ligands were shown to be nearly as potent as Chlo and Cyan in inhibition of [^3^H]Chlo binding and stimulation of [^3^H]Ry binding in house fly thoracic muscle RyR. Although the newly synthesized compounds had only moderate activity in insect larvicidal activity assays, their high *in vitro* potency in a validated insect RyR binding assay encourages further development of fluorescent probes for insect RyRs.

## 1. Introduction

**Figure 1:**
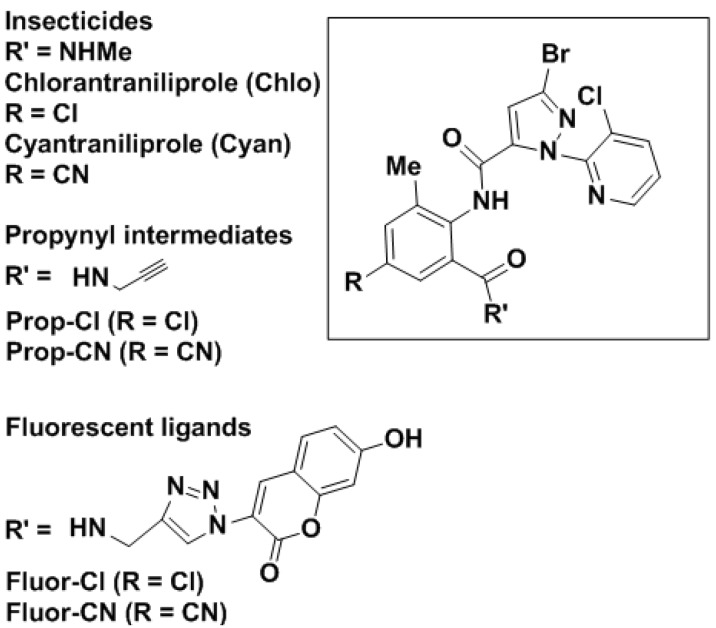
Anthranilic diamide skeleton common to two major commercial insecticides and four analogs with *N*-propynyl and *N*-hydroxycoumarin substituents.

Natural ryanodine (Ry) from *Ryania speciosa* was used as a botanical insecticide in the 1940s [1]. By the late 1990s, two types of diamides were found to possess excellent insecticidal activity, producing poisoning signs similar to those of Ry. Radioligand binding and Ca^2+^ flux studies at Nihon Nohyaku and Bayer [2,3,4,5,6,7,8,9] and at DuPont [10,11,12,13] defined the mode of action of the diamides as Ry receptor (RyR) modulators keeping Ca^2+^ channels open and depleting Ca^2+^ stores, leading to gradual muscle contraction and paralysis. RyRs form intracellular Ca^2+^ channels located mostly on the sarcoplasmic reticulum of muscle. With commercialization of the anthranilic diamides chlorantraniliprole (Chlo) [11] and cyantraniliprole (Cyan) [14] (Figure 1) and phthalic diamide flubendiamide (Flu) [2], the insect RyR became one of the most important insecticide targets. Photoaffinity labeling revealed that Flu interacts in the insect transmembrane domain and that the *N*-terminus plays an important role in sensitivity [8]. Radioligand binding assays with [^3^H]Chlo and [^3^H]Ry established that anthranilic diamides and Ry act at the *Musca domestica* RyR [15,16]. The genes encoding the RyRs have been characterized for various species (*Heliothis virescens, Myzus persicae, Aphis gossypii, Peregrinus maidis* and *Drosophila* *melanogaster*) [10], and more recently for *Plutella xylostella* [17]. However, up to now, very little is known about the structure of the insect tetrameric transmembrane RyRs and particularly their active binding site.

This study takes the first steps in developing a fluorescent probe for the insect RyR. Chlo and Cyan were selected as prototypes because they are exceptionally potent and readily recognizable insecticides. 7-Hydroxycoumarin was used as the fluorescent substituent to be introduced. Retention of the required biological properties was established by high potency in a validated insect (*Musca*) RyR radioligand binding assay and moderate larvicidal activity on an important pest (*Spodoptera*). 

## 2. Results and Discussion

### 2.1. Fluorescent Ligands Design and Synthesis

**Scheme 1:**
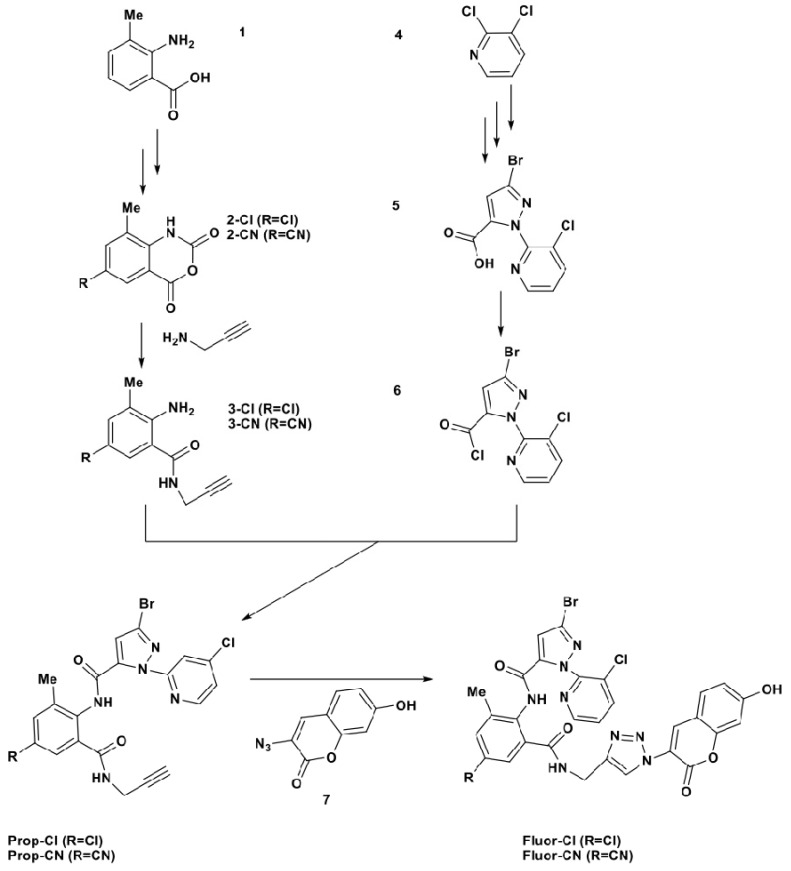
Synthesis routes for two *N*-propynyl intermediates and two fluorescent ligands.

The key step in preparing the effective fluorescent ligands was solved by coupling of Chlo and Cyan as the pharmacophore skeletons and hydroxycoumarin as the fluorescent chromophore with a click reaction (Scheme 1). Both fluorescent ligands obtained are very stable and possess good fluorescent properties, for instance, both their maximum excitation wavelength 396 nm and the maximum emission wavelength 475 nm and their large stokes shift are suitable for fluorescence assay.

### 2.2. Fluorescent Ligands Bind Potently to Specific Site in Musca RyR

**Figure 2:**
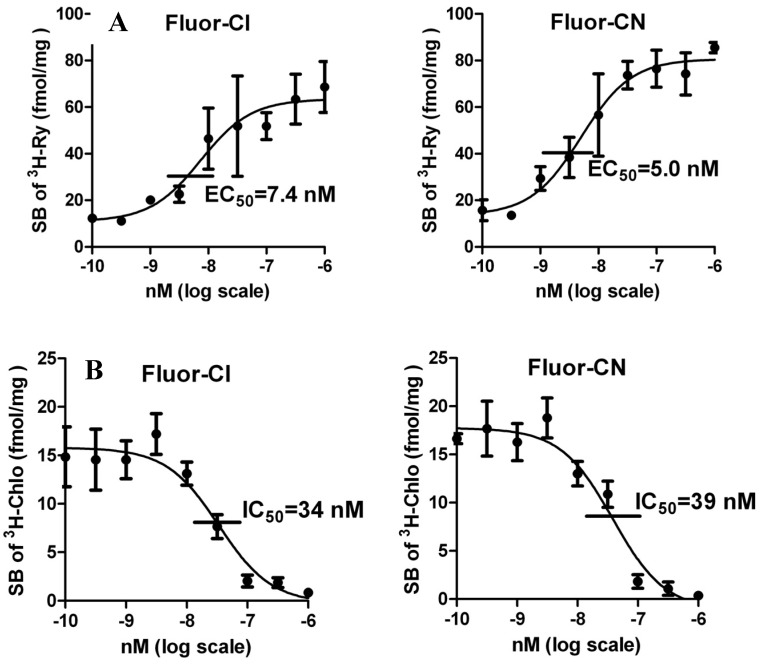
Effects of fluorescent ligands on specific binding of [^3^H]Ry and [^3^H]Chlo in *Musca* muscle membrane. (**A**) Stimulation of [^3^H]Ry binding (EC_50_). (**B**) Inhibition of [^3^H]Chlo binding (IC_50_).

The validated *Musca* RyR assay with [^3^H]Chlo and [^3^H]Ry was used to compare the potencies of the new compounds with the standards Chlo and Cyan. The results showed that Fluor-Cl and Fluor-CN stimulated [^3^H]Ry binding with EC_50_’s of 5–7 nM (Figure 2A) consistent with the reported value of 3–15 nM for Chlo and Cyan [15]. In addition, Fluor-Cl and Fluor-CN inhibited [^3^H]Chlo binding with similar IC_50_’s of 34–39 nM (Figure 2B), compared to 6–14 nM for Chlo and Cyan [15], which indicated that they all work at the same binding site on *Musca* RyRs. 

### 2.3. Insecticidal Activity of Fluorescent Ligands

The results of assays with *Plutella* larvae are shown in Table 1. Although the two N-propynyl compounds were less potent than Chlo, Cyan and Flu, they all showed good insecticidal activity with LC_50_s for Prop-Cl and Prop-CN of 0.08 ppm and 0.34 ppm, respectively. After incorporating the hydroxycoumarin fluorophore, the ligands were 22–32 fold less potent than the propynyl compounds, with LC_50_s of 2.6–7.6 ppm. Thus the new compounds retain good insecticidal activity despite the propynyl and large polar fluorescent substituents were introduced. Most importantly the fluorescent probes are very potent *in vitro* in binding assays, although less toxic *in vivo* with penetration barriers.

**Table 1:** Insecticidal activity for *Plutella* larvae.

Compd	LC_50_ (95%CL) ^a^ (ppm)	Slope SE	*x*^2^ (*df*) ^b^
**Chlo**	0.033(0.006–0.059)	2.5 ± 0.5	13.7(6)
**Cyan**	0.061(0.035–0.086)	5.0 ± 1.0	14.5(8)
**Flu**	0.056(0.037–0.068)	6.9 ± 2.1	1.7(7)
**Prop-Cl**	0.080(0.024–0.152)	1.5 ± 0.3	5.5 (7)
**Prop-CN**	0.34(0.24–0.43)	4.6 ± 1.1	7.9(8)
**Fluor-Cl**	2.59(1.69–3.46)	4.0 ± 0.9	5.2(8)
**Fluor-CN**	7.57(4.57–11.77)	2.4 ± 0.7	3.8(8)

^a^ 95% confidence limits, *n* = 30; ^b^ Chi-square value and degrees of freedom (*df*) as calculated by POLOPlus.

## 3. Experimental

### 3.1. General Methods and Materials

Commercial reagents were used as obtained from Sigma-Aldrich (St. Louis, MO, USA), Alfa Aesar (Ward Hill, MA, USA), and J&K (Beijing, China). Technical grade Chlo (95% purity) was obtained from DuPont Agricultural Chemicals Ltd. (Shanghai, China). Sources for Cyan and other relevant chemicals were reported earlier [15,16]. Hepes was produced in-house. Nuclear magnetic resonance (NMR) spectra were recorded with a 300 MHz spectrometer (Bruker, Billerica, MA, USA) and mass spectra (MS) with a time-of-flight spectrometer (Agilent Technologies, Inc., Santa Clara, CA, USA). Fluorescence intensity was measured with a Cary Eclipse Fluorescence Spectrophotometer (Agilent). 6-Chloro-8-methyl-1*H*-benzo[d][1,3]oxazine-2,4-dione (**2-Cl)**, 8-methyl-2,4-dioxo-2,4-dihydro-1*H*-benzo[d][1,3]oxazine-6-carbonitrile (**2-CN)** and 3-bromo-1-(3-chloropyridin-2-yl)-1*H*-pyrazole-5-carboxylic acid (**5**) were prepared from compounds **1** and **4**, respectively, by the methods of Chai *et al.* [18] and 3-azido-7-hydroxy-2H-chromen-2-one (**7**) was synthesized according to Sivakumar *et al.* [19]. The structures of compounds are shown in Scheme 1.

### 3.2. Synthesis of Propynyl Intermediates

*3-Bromo-1-(3-chloropyridin-2-yl)-1H-pyrazole-5-carbonyl chloride* (**6**). Compound **5** (3.66 g, 12 mmol, 1 eq) was placed in a 100-mL round-bottomed flask, and SOCl_2_ (4.43 g, 37.5 mmol, 3.12 eq) was added dropwise under stirring at room temperature. After addition, the mixture was heated gently to 100 °C and kept for 4 h. The reaction mixture was cooled to room temperature and then excess SOCl_2_was distilled (70 °C) at atmospheric pressure to obtain 3.80 g of compound **6** as a deep colored oil with yield 98%.

*2-Amino-5-chloro-3-methyl-N-(prop-2-ynyl)benzamide* (**3-Cl**). Acetic acid (7.37 g, 44 mM, 3.67 eq) was added to a solution of compound **2-Cl** (6.0 g, 30 mmol, 2.5 eq) and ethyl acetate (50 mL). Propynylamine (2 g, 36 mmol, 3 eq) was added, the reaction mixture was heated and kept at 50 °C for 1 h and then was cooled to room temperature. Water (65 g, 3.6 mol, 300 eq) was added and the solvent was removed by distillation under vacuum. The residue was cooled below 10 °C with an ice bath, forming a precipitate, which was filtered, washed with water (approx. 10 mL), and dried to afford 7.0 g of compound **3-Cl** as a white powder in 96% yield.

*3-Bromo-N-(4-chloro-2-methyl-6-(prop-2-ynylcarbamoyl)phenyl)-1-(3-chloropyridin-2-yl)-1H-pyrazole-5-carboxamide* (**Prop-Cl**). A mixture of compound **6** (9.0 g, 28 mmol, 2.33 eq) and compound **3-Cl** (6 g, 27 mmol, 2.25 eq) in 1,2-dichloroethane (90 mL) was cooled to 10 °C with an ice bath, and the reaction solution was stirred at room temperature for 2 h then *N*,*N*-diisopropylethylamine (4.2 g, 30 mmol, 2.5 eq) was added dropwise. When the reaction was completed, based on thin layer chromatography (TLC) monitoring, water (50 mL) was added and the reaction mixture was stirred for 15 min. The white solid precipitate was collected by filtration, washed with water (3 mL), and dried to afford 9.2 g of compound Prop-Cl as a white powder, yield 92%; m.p. 222.5–223.6 °C, ^1^H-NMR (DMSO-*d_6_*): *δ* 10.24 (s, 1H), 8.79 (t, *J = 5.4* Hz, 1H), 8.49 (dd, *J =* 4.7*,* 1.5 Hz, 1H), 8.16 (dd, *J =* 8.1, 1.5 Hz, 1H), 7.60 (dd, *J =* 8.1, 4.7 Hz, 1H), 7.50 (d, *J* = 2.0 Hz, 1H), 7.38 (s, 1H), 7.33 (d, *J* = 2.3 Hz, 1H), 3.93 (dd, *J* = 5.4, 2.5 Hz, 2H), 3.09 (t, *J* = 2.5 Hz, 1H), 2.16 (s, 3H); ^13^C-NMR (DMSO-*d_6_*): *δ* 165.81, 156.05, 148.85, 147.51, 139.81, 139.67, 139.44, 135.70, 132.07, 131.97, 131.46, 128.31, 127.22, 127.00, 126.00, 111.21, 81.12, 73.55, 28.98, 18.11; HRMS (ESI) calcd for C_20_H_14_BrCl_2_N_5_O_2_ [M+H]^+^ 507.9786, found 507.9806.

*2-Amino-5-cyano-3-methyl-N-(prop-2-ynyl)benzamide* (**3-CN**) *and 3-bromo-N-(4-cyano-2-methyl-6-(prop 2-ynylcarbamoyl)phenyl)-1-(3-chloropyridin-2-yl)-1H-pyrazole-5-carboxamide* (**Prop-CN**). These compounds were synthesized according to the procedures for compounds **3-Cl** and **Prop-Cl** respectively. **Prop-CN**: a white powder, yield 90%; m.p. 223.7–225.0 °C, ^1^H-NMR (DMSO-*d_6_*): *δ* 10.49 (s, 1H), 8.89 (t, *J* = 5.4 Hz, 1H), 8.50 (dd, *J* = 4.7, 1.5 Hz, 1H), 8.16 (dd, *J* = 8.1, 1.5 Hz, 1H), 7.90 (d, *J* = 1.3 Hz, 1H), 7.75 (d, *J* = 1.5 Hz, 1H), 7.61 (dd, *J* = 8.1, 4.7 Hz, 1H), 7.40 (s, 1H), 3.94 (dd, *J* = 5.3, 2.6 Hz, 2H), 3.11 (t, *J* = 2.5 Hz, 1H), 2.21 (s, 3H); ^13^C-NMR (DMSO-*d_6_*): *δ* 165.58, 155.88, 148.75, 147.56, 139.73, 139.54, 138.44, 137.73, 135.99, 134.58, 130.20, 128.29, 127.28, 127.07, 118.41, 111.45, 109.81, 80.94, 73.77, 29.08, 18.14; HRMS (ESI) calcd for C_21_H_14_BrClN_6_O_2_ [M+H]^+^ 499.0130, found 499.0144.

### 3.3. Synthesis of Fluorescent Ligands

*3-Bromo-N-(4-chloro-2-((1-(7-hydroxy-2-oxo-2H-chromen-3-yl)-1H-1,2,3-triazol-4-yl)me-thylcarbamoyl)-6-methylphenyl)-1-(4-chloropyridin-2-yl)-1H-pyrazole-5-carboxamide* (**Fluor-Cl**). To a solution of **Prop-Cl** (0.25 g, 0.5 mmol) and compound **7** (0.1 g, 0.5 mmol) in water and THF (10 mL, v/v = 1:1), freshly prepared aqueous sodium ascorbate (150 μL, 0.15 mmol) and aqueous copper (II) sulfate pentahydrate (125 μL, 0.038 mmol) were added in order. The heterogeneous mixture was stirred vigorously overnight in the dark at room temperature. The solvent THF was removed by distillation, the residue was diluted with water (5 mL), cooled in an ice bath, and then the precipitate was collected by filtration, washed with cold water (10 mL) followed by drying under vacuum to afford 0.3 g of pure **Fluor-Cl** as a yellow powder, yield 85.7%; m.p. 212.3–213.8 °C, ^1^H-NMR (DMSO-*d_6_*): *δ* 10.90 (s, 1H), 10.27 (s, 1H), 8.96 (t, *J* = 5.8 Hz, 1H), 8.53 (s, 1H), 8.44 (dd, *J* = 4.7, 1.5 Hz, 1H), 8.34 (d, *J* = 10.4 Hz, 1H), 8.13 (dd, *J* = 8.1, 1.5 Hz, 1H), 7.75 (d, *J* = 8.6 Hz, 1H), 7.57 (dd, *J* = 8.1, 4.7 Hz, 1H), 7.50 (d, *J* = 1.8 Hz, 1H), 7.39 (d, *J* = 2.3 Hz, 1H), 7.28 (s, 1H), 6.91 (dd, *J* = 8.5, 2.2 Hz, 1H), 6.86 (d, *J* = 2.1 Hz, 1H), 4.48 (d, *J* = 5.5 Hz, 2H), 2.16 (s, 3H); ^13^C-NMR (DMSO-*d_6_*): *δ* 166.22, 162.84, 156.69, 156.16, 155.10, 148.75, 147.41, 145.39, 139.74, 139.65, 139.38, 136.59, 136.24, 131.96, 131.83, 131.49, 131.40, 128.19, 127.16, 126.96, 125.94, 124.19, 119.77, 114.71, 111.02, 110.84, 102.60, 67.47, 18.07; HRMS (ESI) calcd for C_29_H_20_BrCl_2_N_8_O_5_ [M+H]^+^ 711.0119, found 711.0118.

*3-Bromo-1-(3-chloropyridin-2-yl)-N-(4-cyano-2-((1-(7-hydroxy-2-oxo-2H-chromen-3-yl)-1H-1,2,3-triazol-4-yl)methylcarbamoyl)-6-methylphenyl)-1H-pyrazole-5-carboxamide* (**Fluor-CN**). This compound was synthesized according to the procedure for **Fluor-Cl**. A yellow powder, yield 71.4%, m.p. 234.7–236.9 °C ^1^H-NMR (DMSO-*d_6_*): *δ* 10.90 (s, 1H), 10.53 (s, 1H), 9.05 (t, *J* = 5.6 Hz, 1H), 8.54 (s, 1H), 8.45 (dd, *J* = 4.7, 1.3 Hz, 1H), 8.39 (s, 1H), 8.13 (dd, *J* = 8.1, 1.3 Hz, 1H), 7.90 (s, 1H), 7.82 (d, *J* = 1.4 Hz, 1H), 7.75 (d, *J* = 8.6 Hz, 1H), 7.58 (dd, *J* = 8.1, 4.7 Hz, 1H), 7.32 (s, 1H), 6.92 (dd, *J* = 8.5, 2.2 Hz, 1H), 6.86 (d, *J* = 2.1 Hz, 1H), 4.50 (d, *J* = 5.5 Hz, 2H), 2.22 (s, 3H); ^13^C-NMR (DMSO-*d_6_*): *δ* 165.63, 162.55, 156.37, 155.68, 154.79, 148.34, 147.14, 144.93, 139.39, 139.19, 138.09, 137.32, 136.35, 135.54, 134.83, 131.08, 129.88, 127.86, 126.91, 126.70, 123.94, 119.44, 118.14, 114.41, 110.99, 110.51, 109.51, 102.29, 79.31, 17.78; HRMS (ESI) calcd for C_30_H_19_BrClN_9_O_5_ [M+H]^+^ 702.0461, found 702.0449.

### 3.4. Insecticidal Activity

The diamondback moth, (*Plutella xylostella*) was reared in the laboratory for over ten years using vermiculite cultured radish (*Raphanus sativusL. var. cuiqing*) seedlings. Bioassays were conducted using a leaf-dip method slightly adapted from the methods of Liang *et al.* [20] and He *et al.* [21]. Cabbage (*Brassica oleracea* variant L.) leaves measuring 6 × 6 cm were immersed for 10 s in various concentrations of test compound prepared with distilled water containing 1 g L^-1^ Triton X-100. Each leaf was left to air dry for 1.5 h and then placed into a Petri dish lined with filter paper. A total of 15 first day fourth instar larvae was introduced into each dish, and three replicates were prepared. Five to seven concentrations of test compound and one control (distilled water with 1 g/L Triton X-100) were examined in each bioassay. Mortality was assessed after 96 h of exposure as individuals that did not move when pushed gently with a brush. The LC_50_ value was calculated using PoLoPlus 2.0 software (LeOra Software, Petaluma, CA, USA) with data corrected for control mortality by Abbott’s formula [22].

### 3.5. RyR Radioligand Assay

[^3^H]Ry (95 Ci/mmol, Perkin-Elmer Life Sciences, Boston, MA, USA) and [^3^H]Chlo (78 Ci/mmol) were used in RyR binding assays with house fly thorax muscle membranes as reported earlier [15]. Adults emerging from pupae obtained from Benzon Research (Carlisle, PA, USA) were used to collect the thoraces for membrane preparation. The following two buffers were used: (A) 10 μM phenylmethanesulfonyl fluoride, 0.8% bovine serum albumin, and 303 mM sucrose in 20 mM Tris-maleate, pH 7.0; and (B) 0.8 mM CaCl_2_, 2 mM ATP·Mg^2+^ salt, and 1.5 M KCl in 10 mM Hepes, pH 7.4. Incubation mixtures were prepared by sequential addition to culture tubes of 200 μL of buffer B, 200 μL of buffer A containing thorax muscle membranes (200 μg protein) and then ethanol (5 μL) containing [^3^H]Ry or [^3^H]Chlo to give a final radioligand concentration of 1 nM. Finally, test compounds were added in 5 μL ethanol to give the specified concentrations of Fluor-Cl and Fluor-CN. Following incubation for 2 h at 37 °C, the mixtures were filtered through GF/B filters (Whatman, presoaked in ice-cold washing buffer) immediately after dilution with 5 mL of ice-cold washing buffer (150 mM KCl, 10 mM Hepes, pH 7.4). Each assay tube was further rinsed twice with 5 mL of ice-cold washing buffer, and the rinses were passed through the same filter. The filters were then transferred to scintillation vials containing 10 mL of Safety-Solve (Research Products International Corporation, Mount Prospect, IL, USA) and held overnight in the dark before scintillation counting. Binding data were analyzed and plotted by GraphPad Prism 5.0. All data reported are mean ± standard error for two independent experiments with triplicate samples.

## 4. Conclusions

The goal to prepare a highly potent and specific fluorescent probe for the insect RyR has been achieved with an anthranilic diamide analog of Chlo containing a hydroxycoumarin substituent. In a validated *Musca* RyR assay Fluor-Cl is similar to Chlo in potency for stimulating [^3^H]Ry binding and inhibiting [^3^H]Chlo binding all in the range of 5–39 nM. The fluorescent probes are less active than Chlo and Cyan as insecticides as expected for compounds with large polar substituents affecting transport and stability. Large species differences are evident in RyR ligand binding with house fly and honeybee quite sensitive [15,16] and therefore possibly preferred RyR sources. Replacing radioligands with fluorescent probes will allow broader use of binding assays without restrictions associated with radioactive materials. 

## Supplementary Materials

Supplementary materials can be accessed at: http://www.mdpi.com/1420-3049/19/4/4105/s1.
