# Supplementary material for: Fluorescent Probes for Insect Ryanodine Receptors: Candidate Anthranilic Diamides
Source: Molecules. 2014 Apr 2;19(4):4105–14. doi: 10.3390/molecules19044105 (PMC6270845; doi:10.3390/molecules19044105)

Supporting Information

Figure S1. NMR Spectra for the new compounds.

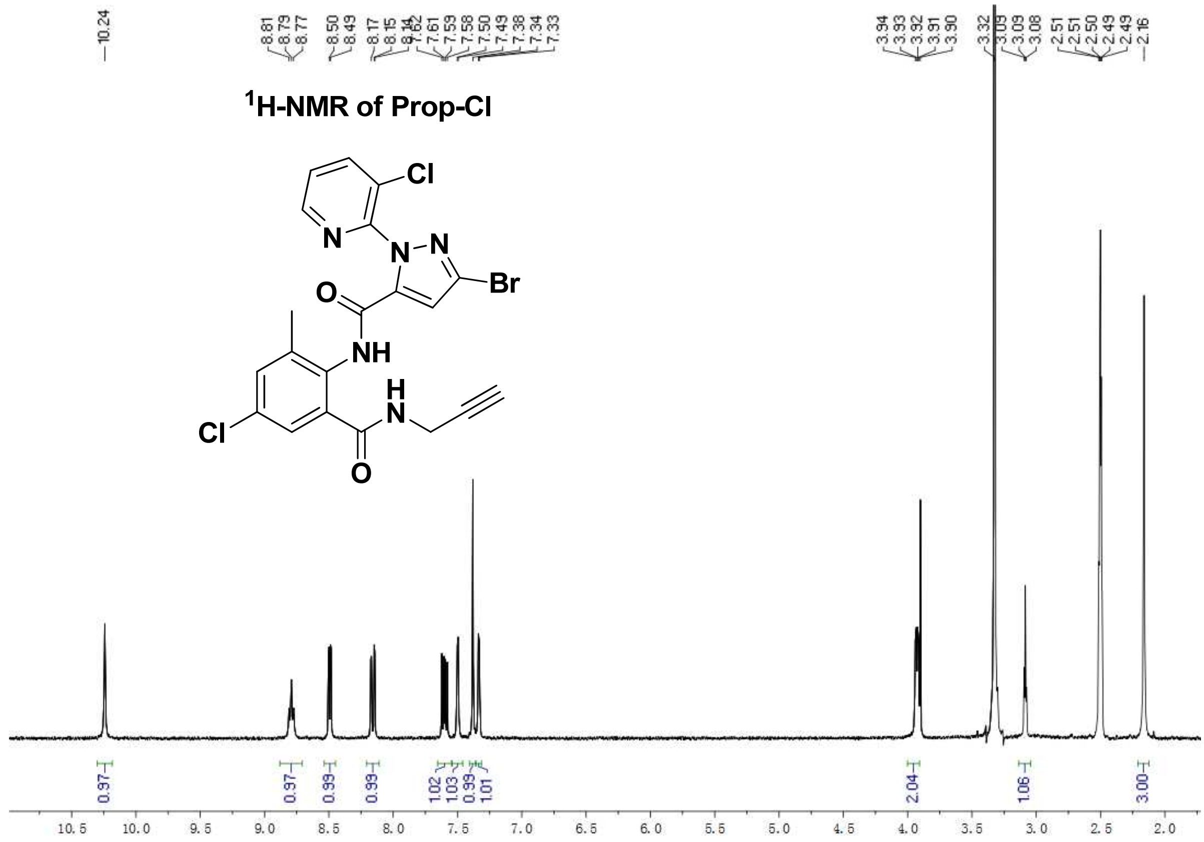

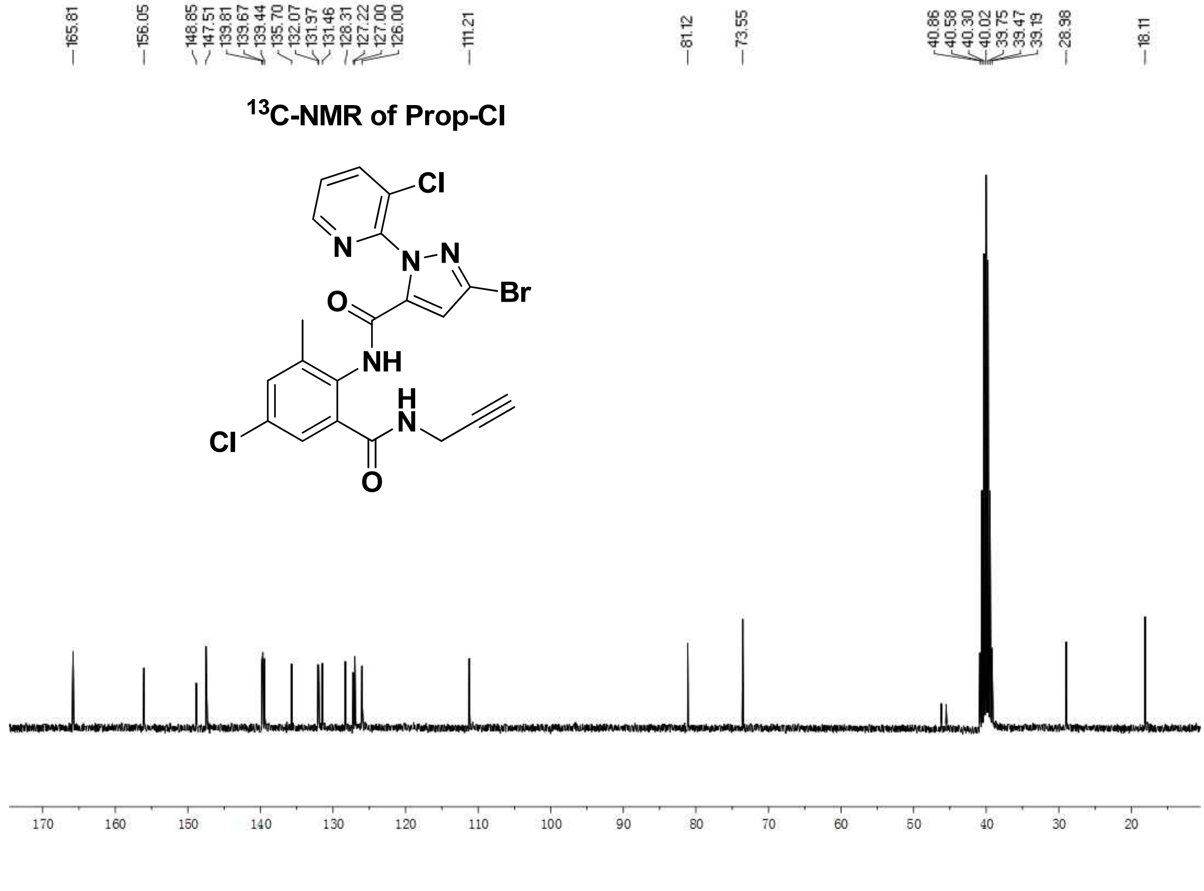

Figure S1. *Cont.*

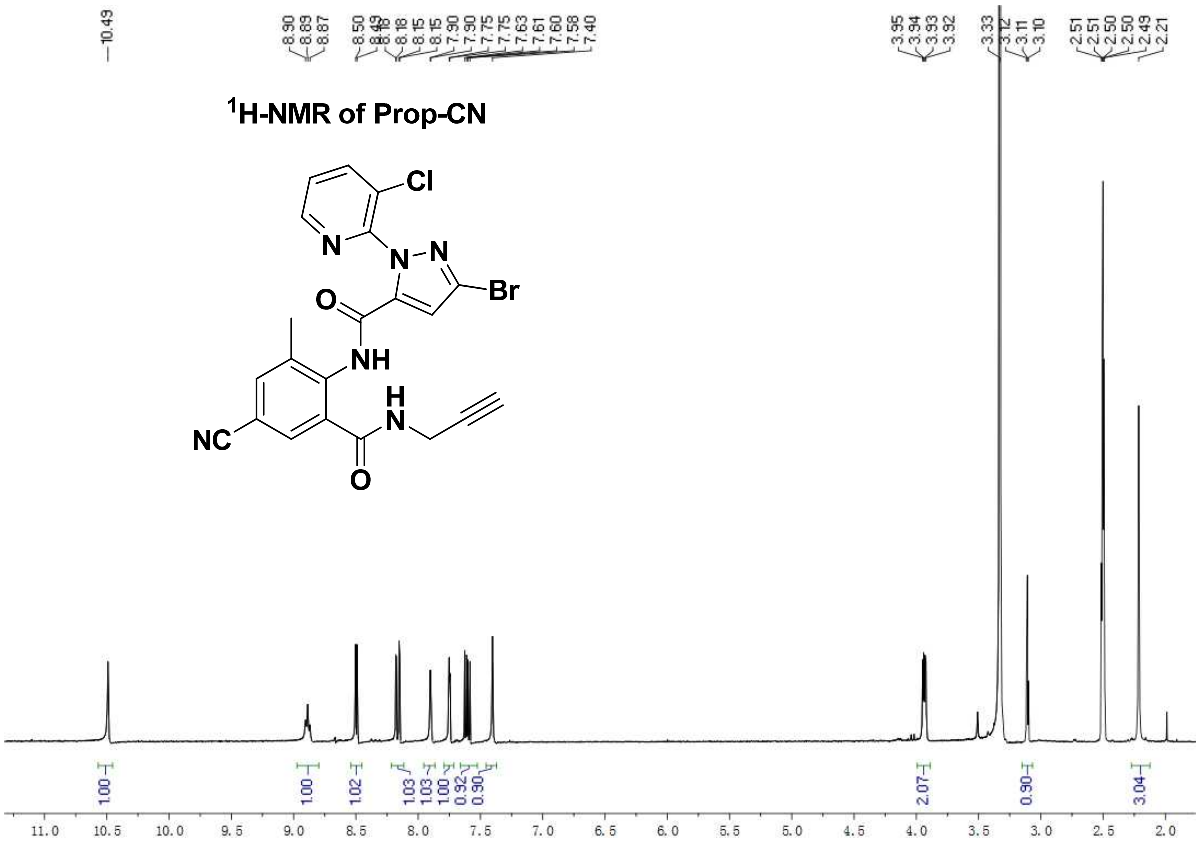

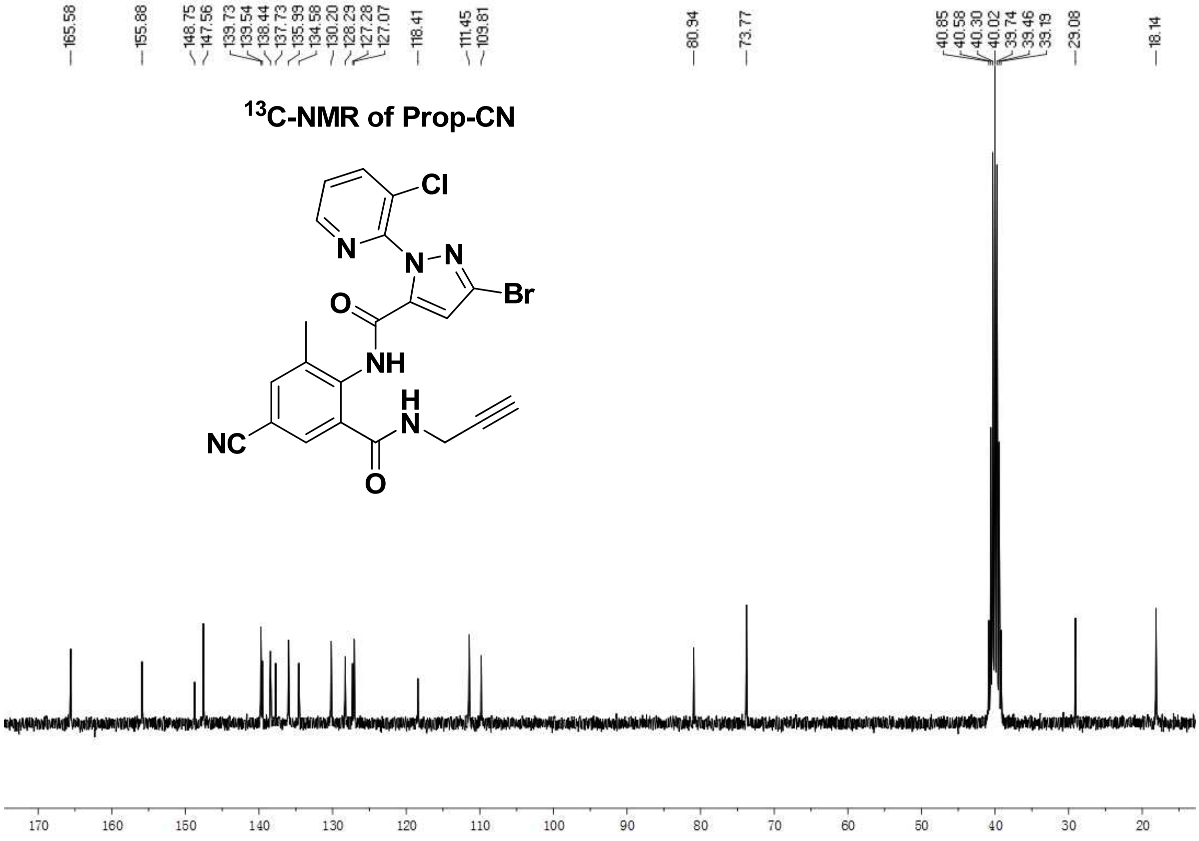

Figure S1. *Cont.*

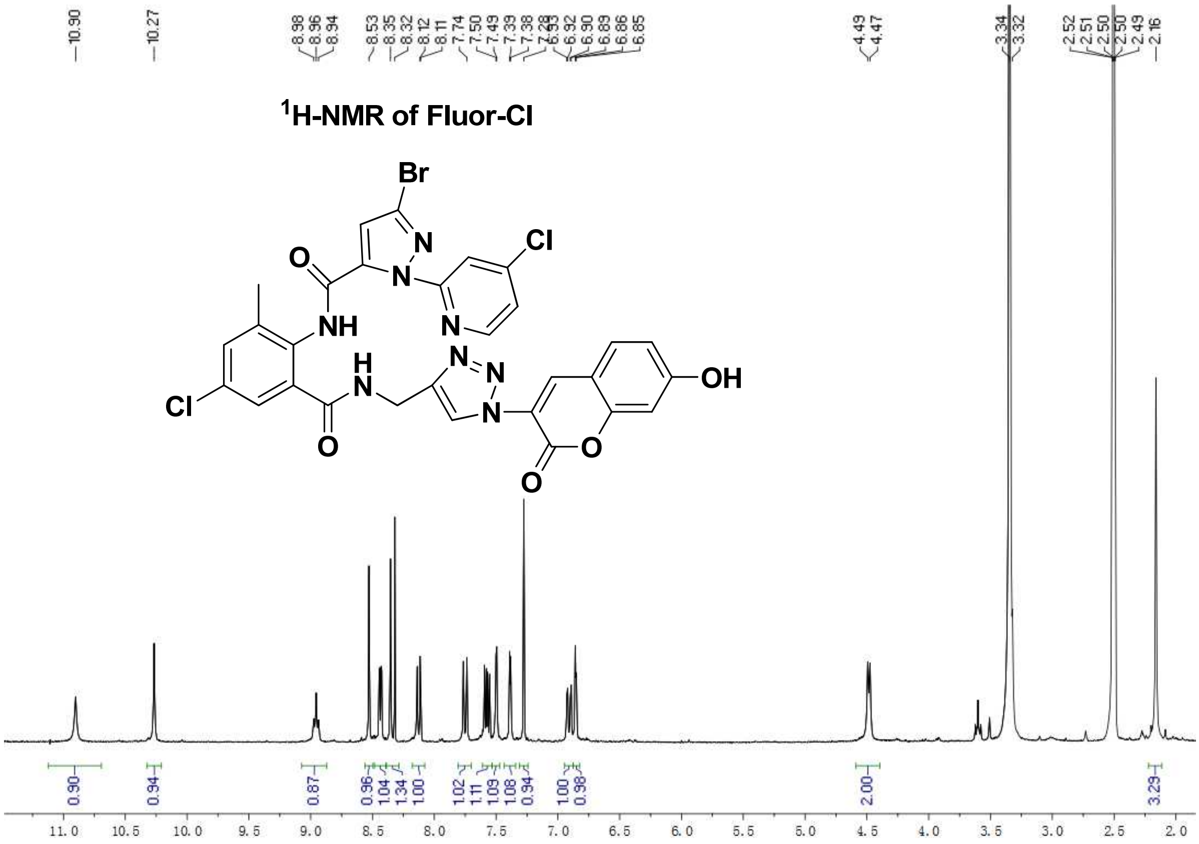

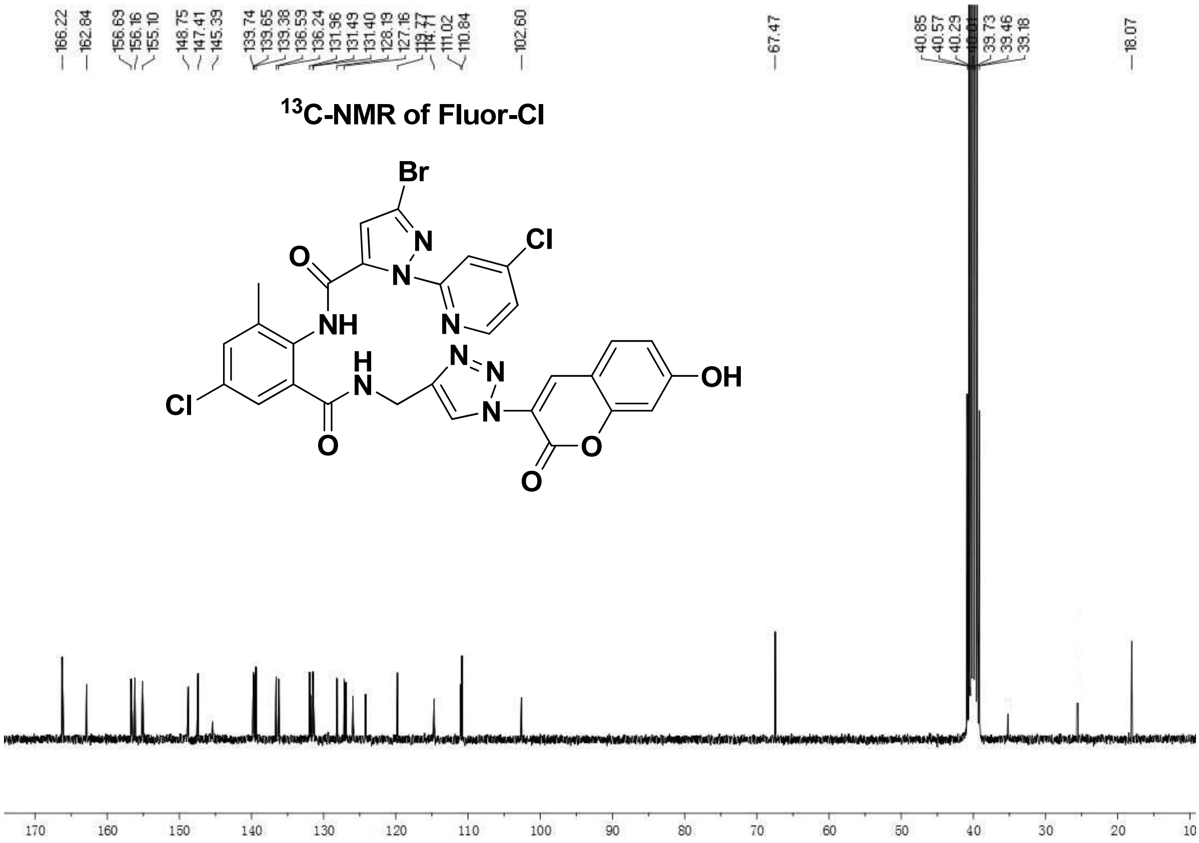

Figure S1. *Cont.*

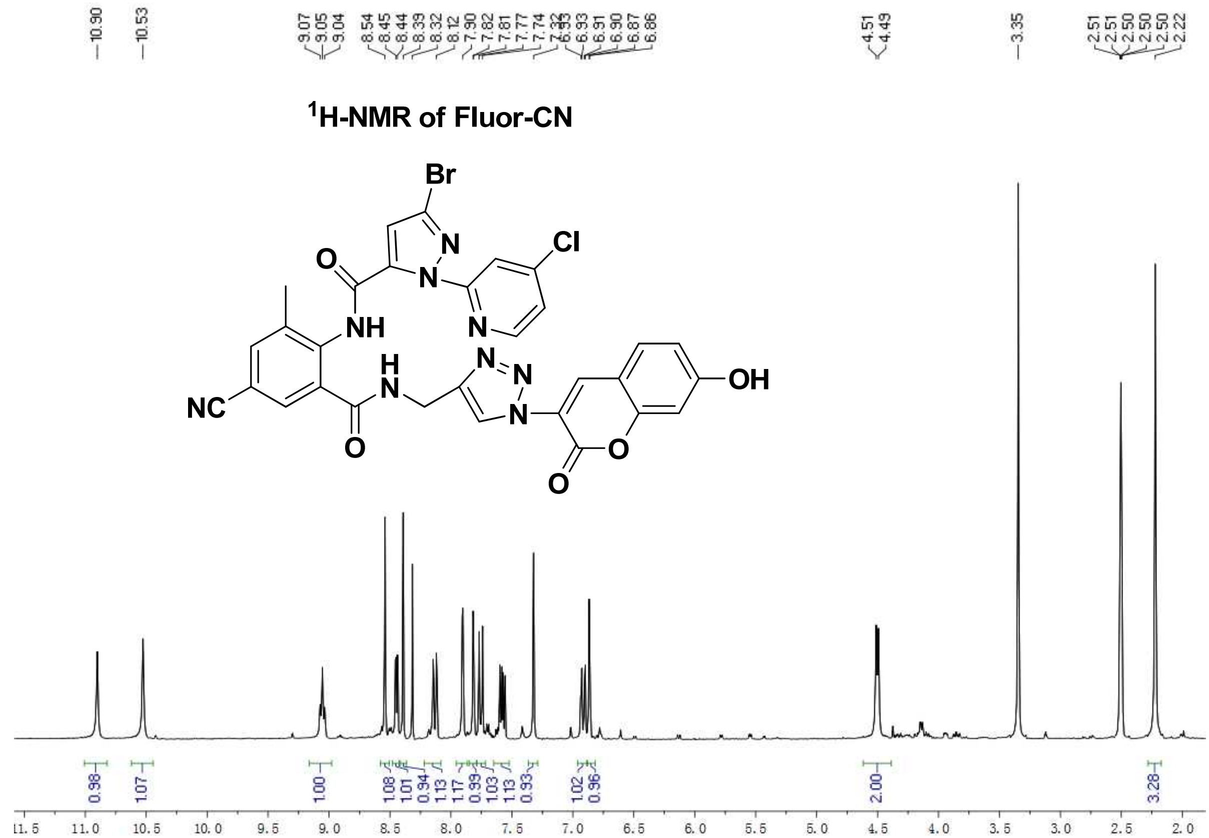

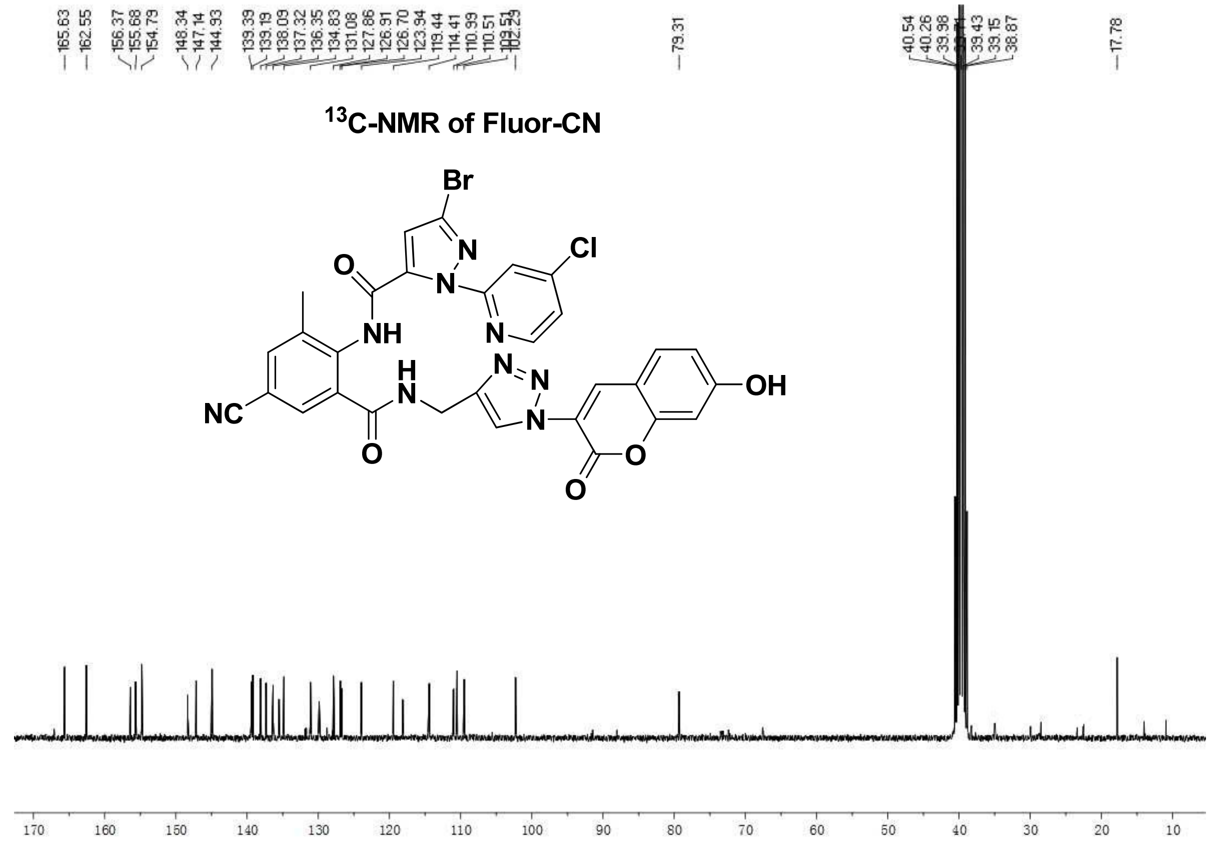

Supplement: Supplementary file 1 [file molecules-19-04105-s001.pdf]
